# Next-generation sequencing: recent applications to the analysis of colorectal cancer

**DOI:** 10.1186/s12967-017-1353-y

**Published:** 2017-12-08

**Authors:** Filippo Del Vecchio, Valentina Mastroiaco, Antinisca Di Marco, Chiara Compagnoni, Daria Capece, Francesca Zazzeroni, Carlo Capalbo, Edoardo Alesse, Alessandra Tessitore

**Affiliations:** 1 Division of Cancer Sciences, University of Southampton, Southampton, Hampshire, SO16 6YD UK; 2 Department of Biotechnological and Applied Clinical Sciences, University of L’Aquila, L’Aquila, 67100 Italy; 3 Department of Medicine, Centre for Cell Signaling and Inflammation, Imperial College London, London, W12 0NN UK; 4 Department of Molecular Medicine, La Sapienza University, Rome, 00161 Italy

**Keywords:** Next-generation sequencing, Colorectal cancer, Precision medicine, Targeted therapy

## Abstract

Since the establishment of the Sanger sequencing method, scientists around the world focused their efforts to progress in the field to produce the utmost technology. The introduction of next-generation sequencing (NGS) represents a revolutionary step and promises to lead to massive improvements in our understanding on the role of nucleic acids functions. Cancer research began to use this innovative and highly performing method, and interesting results started to appear in colorectal cancer (CRC) analysis. Several studies produced high-quality data in terms of mutation discovery, especially about actionable or less frequently mutated genes, epigenetics, transcriptomics. Analysis of results is unveiling relevant perspectives aiding to evaluate the response to therapies. Novel evidences have been presented also in other directions such as gut microbiota or CRC circulating tumor cells. However, despite its unquestioned potential, NGS poses some issues calling for additional studies. This review intends to offer a view of the state of the art of NGS applications to CRC through examination of the most important technologies and discussion of recent published results.

## Background

Since the application of modern technology in medicine, scientists always tried to understand the real nature of nucleic acids. To this end, a great innovation was brought by the pioneer work of Sanger et al. in the late 70s, when they elaborated the most used method to sequence DNA [1], still considered as the gold standard in molecular diagnostics, even though it is expensive and time-consuming. Growing efforts have been made to widen technical knowledge in this field until the discovery of the “second and third-generation sequencing” methods [2, 3]. They are both part of the next-generation sequencing (NGS) technology, a group of techniques revolutionizing the standard concept of nucleic acids sequencing. The great success of NGS technology is due to the capability of massively sequencing millions of DNA reads, with the possibility to perform, at least, multi-gene analysis, by using very low amount of nucleic acids. NGS technology is suitable for rapid and efficient sequencing of complex genomes too, with consequent time and cost reduction. Furthermore, it can also count on a noteworthy flexibility: in fact, its application has been reported to be successful in different research fields such as molecular diagnostics of genetic diseases, infectious diseases, cancer and pharmacogenomics [4–7].

Many studies on cancer took advantage of the use of this technology, due to the possibility to detect high numbers of variants, related to complicated mechanisms of oncogenesis and tumor heterogeneity [8, 9]. Today, molecular profiling of tumors can provide information about diagnosis, prognosis, prediction to therapy response, which can drive clinical decision making. Concerning the precision care in cancer, the eligibility for targeted drugs, able to specifically inhibit aberrantly dysregulated mechanisms, is strictly affected by the “mutational status” of specific genes. The adoption of this type of medication refers to FDA-approved drugs for a specific tumor type, off-label therapies for specific molecular lesions in a non-approved tumor type or to clinical trials focused on analyzing the effects of agents based on the presence of well-defined molecular alterations. In this context, deep molecular characterization of tumors is a key element for optimal patient’s management and unified guidelines to detect and classify variants, interpret and report results should be recommended and adopted [10].

Colorectal cancer (CRC) is one of the most interesting fields of NGS application. CRC is among the most lethal and frequent types of tumors in the industrialized world and is characterized by a great quantity of activating mutations [11]. These features are the reason why the number of studies employing next-gen techniques is rising in the last years. Their use led to the production of significant results about the identification of novel mutations/altered genes or genomic rearrangements and the possible evaluation of therapy response. This review is intended to condense the state of the art about next-gen technologies and their applications in CRC throughout the last years to provide a useful synopsis. We describe and discuss: (i) NGS main applications and platforms, (ii) main features of CRC pathogenesis and therapy, (iii) NGS application in CRC, by taking into consideration diagnostics of actionable genes, novel mutations, less frequently mutant genes, transcriptomics, epigenetics alterations, other possible directions. Finally, we present some concluding remarks.

## NGS technology

### NGS applications

Next generation sequencing technologies are increasingly used in many fields. Their power consists in the possibility to obtain huge amount of data and discover novel and essential information about the human genome. This feature opened many contexts of successful applications. The first was whole-genome sequencing (WGS), an approach intended for entire genome sequencing. It provides the most complete landscape of genomic information and possible biological consequences [12]. Despite its potential, which permits the discovery of undescribed mutations at the level of coding as well as non-coding regions, mostly involved in the regulation of gene expression, it shows undeniable difficulties due to the high amount of generated data and their validation (i.e. variants with unknown significance—VUS-, intronic mutations, etc.). Furthermore, the method requires considerable human resources for efficient organization and interpretation [13]. To avoid these difficulties and obtain at the same time extensive high-throughput data, less labored methods, such as whole-exome sequencing (WES), targeted sequencing [14] or transcriptome sequencing (RNA-Seq), have been conceived. The first one provides only information about exons, restricting the length of the nucleic acid analyzed within coding regions; whereas targeted sequencing is focused on specific subsets of regions, or more relevant genes, whose pathogenic involvement in specific diseases has already been described or supposed. Transcriptome analysis generates data about splicing variants, allelic expression [15], RNA editing [16] and alternative 3′-UTR polyadenylation as well [17]. Finally, the study of epigenetic modifications is becoming an emerging field of NGS application, particularly in cancer research. The analysis of elements playing a role in such mechanisms, such as methylated sequences, DNA-binding proteins or non-coding RNAs, could aid in defining profiles that can be used for diagnostic and/or prognostic purposes [13, 18].

### Next-generation platforms

Automated Sanger sequencing is still considered as the “gold standard” in molecular diagnostics. As above mentioned, cancer is a very complex disease, characterized by abnormal functions of multiple genes and sophisticated mechanisms physiologically directed to protect normal cell functions. The entry of new generation technologies in cancer research provided the possibility to perform multi-gene analysis, contributing to shed light on complicated molecular mechanisms of oncogenesis and, more, offering a great contribution at the translational level in the field of the precision medicine. Big companies have developed high-performing instruments able to massively generate hundreds of thousands of sequencing reactions in parallel. These technologies reach good standards of quality and reliability, and are now used in an increasing number of laboratories worldwide for multi-gene or even genome, exome and transcriptome analysis. Basically, they work by using dye terminators, pyrosequencing, monitoring pH changes or sequencing at single-molecule level. Recently, nanopore-based technologies have been applied as well. A brief description of the most common platforms is provided below, along with their main features.

#### Roche

The first NGS technology was released in 2005 with the 454 Genome Sequencer (Life Sciences, today Roche). The great initial success can be attributed to its key point: the association between emulsion PCR, a new amplification strategy, and pyrosequencing. Emulsion PCR is an innovative methodology which uses small water droplets scattered in a lipid solution, where individual DNA fragments are amplified. Briefly, DNA is fragmented, ligated to adapters and mixed to micro-beads containing complementary adapters. DNA fragments-beads complexes are emulsified in droplets containing PCR reactants, so that each droplet contains a single copy of DNA fragment (or a single allele) to be amplified. Afterwards, a standard PCR reaction amplifies DNA. At the end of the procedure, every bead carries on its surface even hundreds of thousands of amplified fragments. Samples are then loaded onto the wells of a picotiter plate to perform many thousands of pyrosequencing (PPi) reactions in parallel by sequentially adding, one at a time, the four deoxynucleotides. A CCD camera detects light signals. Due to the PPi chemical/physical features, this technology can give rise to errors within homopolymeric stretches, with consequent mistakes in the estimated length and introduction of “indel” errors. The two most recent platforms, GS Junior and GS FLX, greatly improved the output, with significant read lengths of 400 and 1000 nucleotides (nt) respectively, close to that reached by Sanger single-gene sequencing, and maximum throughput performance around 700 Mb (Table 1) [19].

**Table 1:** Comparison among the most used NGS platforms

	Roche		Illumina					Thermo Fisher				Pacific Biosciences		Oxford Nanopore	
	GS Junior	GS FLX +	MiniSeq	MiSeq	NextSeq	HiSeq/HiSeqX	NovaSeq	SOLID 5500W	Ion PGM	Ion Proton	Ion S5	RS II	Sequel	MinION	GridION/PromethION
Max output	35 Mb	700 Mb	7.5 Gb	15 Gb	120 Gb	1500/1800 Gb	6000 Gb	320 Gb	1–2 Gb	10 Gb	10–15 Gb	1 Gb	10 Gb	10–20 Gb	50–100 Gb/ Tb scale
Max reads/run	0.1 M	1 M	50 M (PE)	50 M (PE)	800 M (PE)	3 G (PE)	20 G	1.4 G	5.5 M	60–80 M	3–80 M	50 Kb	500 Kb	2–4 M	14–1000 M
Max read length	400 b	1000 b	2 × 150 b	2 × 300 b	2 × 150 b	2 × 150 b	2 × 150 b	50 b	400 b	200 b	600 b	15 Kb (mean read lenght)	15 Kb (mean read lenght)	300 K	300 K
Accuracy (max)	99% at 400 b	99.9% at 15× coverage *E. coli*	99.9% for more than 80–85% of bases	99.9% for more than 75–90% of bases	99.9% for more than 75–80% of bases	99% for more than 75–85% of bases	99.9% for more than 75–85% of bases	> 99%	> 99%	> 99%	99% (expected)	99%	99%	96%	96%
Run time	10 h	10–23 h	7–24 h	4–56 h	12–30 h	1–3.5/<3 days	19–40 h	10 days	2–7 h	2–4 h	2.5 h	0.5–10 h	0.5–10 h	2 min–48h	2 min–48 h
DNA/RNA input^a^	500 ng	200 ng	1–200 ng	1–100 ng	50 ng–1 µg	50 ng–1 µg	100 ng–1 µg	10 ng–5 µg	1–100 ng	1–100 ng	1–100 ng	10 ng–1 µg	10 ng–1 µg	200 ng	200 ng
Max prep time/ Hands-on	24 h	24 h	1 day/ 4 h	7 h	9 h/ 6 h	2.5 days	2.5 days	8 h	10 h/ 4.5 h	10 h/ 45 min	45 min hands-on	6 h/ 4 h	6 h/ 4 h	10 min (1D)	10 min (1D)
Key applications	A	B	C	D	E	F/G	F	H	I	L	M	N	O	P	P

**A** Large–small genome de novo sequencing, resequencing, transcriptome, DNA–protein interaction, methylation, shotgun metagenomics

**B** De novo complex genome assembly, cDNA/transcriptome assembly, sequence capture, shotgun metagenomics

**C** Small whole genome, targeted gene seq, targeted gene expression profiling, miRNA and small RNA

**D** Small whole genome, targeted gene seq, de novo seq, targeted gene expression profiling, miRNA and small RNA, DNA–protein interaction, 16S metagenomic seq

**E** Large and small whole genome, exome, targeted gene seq, de novo seq, whole transcriptome, gene expression profiling with mRNA-Seq, miRNA and small RNA, DNA–protein interaction, methylation, metagenomic seq

**F** Large and small whole-genome, exome, targeted gene seq, whole transcriptome, gene expression profiling, small and miRNA, DNA–protein interaction, methylation, shotgun metagenomics

**G** Human population scale studies, non human whole genome seq

**H** Whole genome/exome sequencing, de novo seq, targeted resequencing, gene expression, microRNA, ChIP, methylation analysis

**I** Targeted DNA-RNA seq, microbial sequencing

**L** Exome-seq, de novo seq, gene expression seq, transcriptome, small RNA seq, ChIP seq

**M** Targeted gene seq, transcriptome, targeted RNA seq, small RNA, de novo microbial sequencing

**N** Whole genome sequencing of small genomes, targeted sequencing, complex population analysis, RNA sequencing of targeted transcripts, microbial, epigenetics

**O** Whole genome de novo assembly, studies for structural variants, transcriptomes, targeted transcript, epigenetic modifications

**P** De novo sequencing, targeted sequencing, metagenomics, epigenetics

*M* million, *PE* paired ends, *Mb* megabases, *Gb* gigabases, *Tb* terabases, *b* bases

^a^Depending on the library type

#### Illumina-solexa

Genome Analyzer, the first Solexa sequencer, was launched in 2006, giving scientists the possibility to analyze 1 Gb of data in a single run. In 2007, the company was acquired by Illumina. The Illumina sequencing technology is based on the use of clonal arrays coupled to clonal massive sequencing by synthesis (SBS) by using cyclic reversible termination (CRT). In brief, after library preparation, DNA fragments, ligated to specific adapters, are provided. Library fragments hybridize to oligos immobilized onto a flow cell and polymerization of a complementary strand occurs. After, the template is washed away and the immobilized complementary single-stranded fragment of new synthesis is in situ amplified by the original mechanism of bridge amplification. This process is repeated to produce billions of clusters which result in clonal amplification of all the fragments. Then sequencing takes place by fluorescently-labeled nucleotides’ incorporation, detected by light source excitation. Identical fragments are massively sequenced in parallel, basecalling is determined by emission wavelength and signal intensity as well. The recent 2-channel technology, which uses a mix, instead of 4, specific dyes, further improved the process, by maintaining the same level of accuracy and reducing the number of image acquisitions per cycle from 4 to 2, with consequent time saving. The complete procedure is directed to sequence forward and reverse strands: therefore, the final analysis considers data from both strands. The applications of Illumina instruments range from genomics to transcriptomics and epigenomics. To meet different needs, the company offers a series of several versatile instruments characterized by different levels of performance (MiniSeq, MiSeq, NextSeq, HiSeq and HiSeq X). The read length ranges from 150 to 300 bp, with more than 99% accuracy (Table 1) [20].

#### Ion Torrent Thermo Fischer

Ion Torrent technology is based on original chemical/physical principles, different from those characterizing the above-mentioned next-generation platforms. Commercialized in 2010 by Life Technologies, it is a semiconductor-based technology where minimal pH changes, produced by the release of hydrogen ions as by-product of nucleotide incorporation, are detected. This is possible by using an “Ion chip”, structured into two parts, to deliver reactants and communicate directly with a proton detector for nucleotide identification during the reaction of incorporation. Different from the other NGS technologies, Ion Torrent recognizes added nucleotides avoiding the use of fluorescence. In fact, the instrument interrogates one nucleotide at a time and incorporation’s specificity is guaranteed by the release and detection of H^+^ ions. In this case, the most frequent errors are caused by phasing. This means that, especially for homopolymer sequences, not all DNA fragments could incorporate nucleotides at each step. Nonetheless, the error rate for this system is very low (i.e. 1%). Continuous improvements have increased read length from the initial 100 to 200 nt (actual average value). Ion Torrent throughput made an even greater jump, starting from 10 Mb to the current maximum of 15 Gb (Table 1) [21].

#### SOLiD

SOLiD are the initials of sequencing by hybridization-ligation implemented in oligonucleotide ligation and detection. This technology was originally developed by Applied Biosystems. The system shares with 454 Roche the emulsion PCR as first amplification step during the DNA library construction. But the very distinctive feature of SOLiD platforms is in the chemistry of its sequencing phases. Amplified fragments are placed on surfaces and subjected to multiple hybridization and ligation reactions according to a fluorescent dye scheme. Each dye labels four dinucleotides for a total of 16 dinucleotides combinations labeled by four different fluorescent dyes. In this way each position is labeled twice, therefore, after two sequential ligation events, the resulting color will identify the incorporated nucleotide. This system is also useful to distinguish incorrect sequence identification from sequence polymorphism. Indeed, the former is detected only in one of the two ligation reactions whereas the latter is detected in both reactions [21, 22].

#### Pacific Biosciences

In 2010, Pacific Biosciences Inc. launched into the market its own innovative platform PacBio, based on the new approach of single-molecule real-time sequencing (SMRT) [23]. Ten of thousands of Zero-Mode Waveguides (ZMWs) chambers, with the smallest light detection volume, in SMRT cells are illuminated from below at the bottom, where DNA polymerase and template are immobilized. After addition of specifically labelled phospho-linked nucleotides, the system detects the incorporation of each of them, revealed by specific fluorescent light emission. The process occurs simultaneously in thousands of ZMWs, providing millions of sequencing reads. Due to these features, the system is exposed to errors caused by missing registration of base incorporation or by wrong interpretation of nucleotide dwelling in the active sites. However, the current error rate is only 0.1%. Concerning read length, PacBio platforms show great performance (up to 20,000 bp read length) up to 10 Gb (Table 1) [24].

#### Oxford Nanopore technologies

In 2012 Oxford Nanopore announced a new technology able to directly sequence a DNA fragment by measuring the change in current flow, due to the passage of such molecule through a nanopore embedded within a membrane [25, 26]. MinION, the main platform today available, is a portable, small (100 g weight), USB powered apparatus provided by a flow cell with 2048 individually addressable nanopores which are controlled, in four groups of 512, by application-specific integrated circuit (ASIC). Briefly, ends of 8–10 Kb genomic/cDNA fragments are ligated to different adapters identified as lead, hairpin and training. Lead adapter promotes capture and loading of a processive enzyme at the 5′ end of a single strand. DNA molecule is captured at the level of an empty nanopore (open channel) and the enzyme activates the strand’s translocation through the pore, ensuring unidirectional single-nucleotide shift. After the hairpin adapter, linking strands each-other, passes through the enzyme, the same process takes place for the complement strand. The passage of trailing adapter leaves the nanopore empty (open channel). As the molecule moves through the nanopore, sensors in the detection system detect changes in ionic current, due to differences in the nucleotide sequence. The current changes are computationally elaborated as a sequence of 3–6 nucleotide long kmers (“words”) using graphical models. The maximum read length is now approaching 1 Mb, with base calling accuracy up to 99%, the time to first usable read is 2 min. GridION X5 and PromethION are more processive platforms which combine multiple MinION devices (Table 1) [27].

#### Qiagen GeneReader

Very recently, Qiagen launched into the market the GeneReader system [28]. This is a standardized NGS workflow designed from nucleic acids’ extraction to insight and specifically directed on analysis of cancer genes’ panel. For this purpose, the QCI Qiagen clinical insight is provided to interpret data and to quickly identify genetic markers associated with approved therapies. The GeneReader platform is based on the SBS technology and is characterized by high scalability and flexible throughput. The system was successfully validated on FFPE CRC samples in comparison to other known platforms [29].

#### Bionano technologies

Bionano genomics put sequencing technologies on new track using the optical mapping concept. Its great innovation is based on the possibility to fluorescently label sequence-specific traits of long, high-molecular weight DNA (up to 1 Mb) to have an optical barcode per each DNA molecule. DNA is then loaded in nanotunnels and channels where it is linearized and imaged by a high-resolution camera. The images are converted into digital label patterns which are de novo assembled by dedicated algorithms to fully reproduce the original genomic map [30]. Due to these features, Bionano genomic mapping technologies allow to increase detection rates of large structural variations and improve assembly contiguity which can be missed because of the too small and fragmented reads generated by conventional NGS sequencing. A recent study confirmed the great potential of this technology in the discovery of novel genetic rearrangements in cancer [31].

### NGS platforms comparison

The possibility to use multiple NGS platforms gave rise to comparisons aimed to evaluate the different characteristics among them. This could help in achieving data to define singular key aspects of the platforms and provide directions about their outputs and when they can better perform. A comparison among the most relevant technical features of most of the instruments previously described is reported in Table 1, where differences are identified in terms of maximum output, reads per run and read length, accuracy, run time, amount of nucleic acids necessary for analysis, experimental time, and key applications. NGS platforms can generate short (SOLID, Ion Torrent, Roche, Illumina) or longer reads (PacBio, Oxford) [32]. Considering the main differences among platforms belonging to each group, Roche and Ion Torrent display higher read lengths with respect to Illumina, but, on the other hand, produce more indel errors, especially within homopolymer regions [33, 34]. Ion Torrent is fast and versatile, making it possible, depending on the necessity, to use different types of chips with specific related yield [21]. Conversely, Illumina is less predisposed to homopolymer errors, it shows an overall accuracy greater than 99.5%, but sometimes can provide under-representation of regions (i.e. AT/GC-rich) and nucleotide substitution errors [35–38]. Both PacBio and MiniONS platforms generate much longer reads and are more suitable for de novo genome assembly or transcript sequencing. PacBio shows high error rate (10–15%), consisting in common indel errors [39], but fortunately they are casually reported within each single sequencing iteration (single-pass). For this reason, the problem may be overcome by using an adequate coverage [40]. MinION is an easily portable device, USB provided, which shows some limitations in the analysis of very long fragments [32]. Due to the technology, providing huge number of distinct signals, it displays large error rate, mainly indels. In addition, homopolymers cannot be accurately sequenced, being difficult precisely distinguish in the nanopore signals due to the same type of “leaving” and “entering” nucleotide [32]. The same limitation appears with modified nucleotides, altering the typical nucleotide-dependent voltage variation.

Several studies have been addressed to compare NGS platforms’ overall performance in various fields. Roche 454, Ion Torrent PGM and MiSeq were in parallel used in metagenomics [41] and differences were highlighted: Ion Torrent resulted unbeatable for speed, Roche 454 produced longer reads, whereas MiSeq provided greater coverage depth and breadth. Quail et al. [42] evaluated the performance of Ion Torrent, PaciBio and Illumina to sequence 4 microbial genomes with CG content ranging from 19.3 to 67.7%. They showed that Ion Torrent detects more variants, but also gives more false-positive results. Moreover, context-specific errors were detected in PGM and MiSeq, but not in PacBio instruments. Another work [43] described the use of Ion Torrent and Illumina HiSeq 2000 for the analysis of a *Rhodobacter* sample with high GC content. Ion Torrent sequencing quality was more stable than HiSeq 2000, where decay of fluorescence signal occurred, and superior in terms of GC depth distribution reproduction. Other published data display higher sensitivity of PacBio RSII technology compared to classical PGM and MiSeq, with identification of mutations in stool DNA at 0.5% frequency [44]. Some papers focused the attention on the strength of different platforms in cancer somatic mutation detection. Misyura et al. [45] compared the performance of MiSeq and Ion Proton by analyzing FFPE samples with amplicon-based commercial panels, consisting of approximately 50 relevant genes in cancer pathogenesis (MiSeq-APC and Proton-CHP). They evidenced 100% concordance in genomic regions subjected to analysis by both panels, including 27 low-frequency (< 15%) variants. Ion Proton, contrary to MiSeq, resulted suitable also for the study of low quality/quantity DNA. This NGS dual approach, characterized by different chemistries, allowed to accurately identify even low-frequency somatic mutations, not detectable by conventional Sanger sequencing. Another group [46] used HiSeq or NextSeq to analyze NGS panels spanning 47 genes (relevant in pheochromocytomas, breast, CRC, renal, pancreatic and ovarian/uterine cancers) in 20,000 hereditary cancers. In this study, almost 8000 non-polymorphic variants were detected and further subjected to validation by Sanger sequencing. Among them, approximately 1.3% of NGS results, mainly located in complex genomic regions (i.e. A/T, C/G rich, homopolymer), were identified by both platforms as false positive. Conversely, simulating zero false-positive rate, the sensitivity of the assay decreased from 100 to 97.8%, with 176 (2.2%) clinically relevant variants detected by Sanger sequencing and missed in NGS. These results, obtained just after processing a huge number of samples, indicated that analysis parameters and threshold levels should be appropriately set-up by a bioinformatics pipeline. In addition, Sanger sequencing should be used to confirm NGS results as well. A comparison between GS-454 and Ion Torrent was provided by Hinrichs et al. [47]. This study was focused on analyzing the most used methods for detecting KRAS/EGFR mutations in 25 FFPE lung cancer samples, already genotyped by Sanger sequencing. Ion Torrent technology (Ampliseq cancer panel) performed better than GS-454 (5 amplicons covering KRAS/EGFR hot spot) which failed in identifying KRAS mutations in four samples.

## Colorectal cancer (CRC)

### CRC: main features

Colorectal cancer (CRC) is referred to tumors affecting colon and rectum and represents the third most common type of tumor worldwide [11]. Although it is included among the worst malignancies, its incidence in the Western countries, particularly in high-income nations, remained almost constant during the last 20 years, whereas it appeared increasing in Eastern countries (Eastern Europe included). CRC are infrequent under 45, being 70% of patients diagnosed over 65 years [48]. Many important progresses have been made for therapeutic procedures, paying attention to the optimization of surgical resection. Diet and physical exercise are two central points in CRC prevention. Studies evidenced that red meat, alcohol and smoke abuse as well as obesity are important risk factors; on the contrary, physical activity seems to protect against the tumor [49, 50]. Additional recognized risk factors are the so-called “inflammatory bowel diseases”: in fact, several studies stated that the presence of an ulcerative colitis or Crohn’s disease implied a greater risk of CRC occurrence [51, 52]. As for other cancers, patients who have been affected by a previous CRC are at risk for developing a second tumor [53].

### Molecular genetics of CRC

Colorectal cancer is a very heterogeneous type of cancer and accounts for either sporadic or hereditary form. Sporadic tumors are more frequent compared to the inherited, which correspond to only 5% of cases [54]. The latter are defined as familial adenomatous polyposis (FAP) [55], hereditary non-polyposis colorectal cancer (HNPCC) [56] and MUTYH associated polyposis (MAP) [57], characterized by the presence of APC, MMR and MYH gene lesions, respectively. CRC was the first type of tumor described in its progression, serving as a model for the characterization of solid tumors’ carcinogenesis. In ‘90s, Fearson and Vogelstein developed the “adenoma-carcinoma sequence” [58], concluding that the progression from an adenomatous polyp to invasive carcinoma is due to sequential acquisition of somatic mutations in different genes (i.e. APC, KRAS, BRAF, SMAD4, TP53). They described deletions on chromosome 5q regions, linked to the adenomatous polyposis coli (APC) gene, and subsequent activating mutations of KRAS gene as associated with the early phases of carcinogenesis. Additional deletions in the adenoma-carcinoma sequence were reported on chromosome 18q, related to deleted in colon cancer (DCC) gene. Then, other deletions at the level of chromosome 17p were detected, with tardive mutations affecting TP53. The general acceptance of this theory by the scientific community led to define colorectal cancer carcinogenesis as a process characterized by multiple sequential mutations.

Genetic instability was considered a leader event in CRC as well, and distinct pathways correlated to the disease have been identified. Chromosomal instability (CIN) accounts for 70–85% of cases and is characterized by accumulation of numerical and structural chromosomal abnormalities or loss of heterozygosity (LOH). This pathway is associated to lesions at the level of several genes, such as APC, KRAS or TP53 genes [59, 60]. Microsatellite instability (MSI) is defined as a hypermutable phenotype, causing a great number of genetic errors, due to mutations affecting genes acting in the repair of the mismatch (MMR) [61]. In addition, dysregulated epigenetic mechanisms have been described [62]: tumors with a CpG island methylator phenotype (CIMP) were reported even in early lesions of the colonic mucosa [63]. Furthermore, it is known that approximately 15% of CRC, mostly sporadic, show also MSI, arising from the MLH1 promoter methylation [64].

### Connections between CRC and gut microbiota

Recently, the existence of an influence exerted by gut microbiota on CRC development has been highlighted, since gut microbiota seems to be directly involved in the regulation of intestinal immune system and promotion of intestinal inflammation. Generally, in healthy conditions, main components of the gut microbiota are represented by obligate aerobic bacteria. When this situation is altered, there is a change in microbiota composition and aerobic bacteria become to be replaced by facultative anaerobic ones. This condition, called “dysbiosis”, is dangerous because intestinal tissue begins to be populated by microbes able to induce inflammatory processes [65]. The creation of an inflamed microenvironment predisposes to carcinogenesis. In this case, a study on mice [66] demonstrated that the integrity of the intestinal barrier is deteriorated with facilitated invasion of tissue by microbes and massive production of cytokines maintaining the inflammatory state. Proinflammatory interleukins, such as IL-17 and IL-23, are released and sustain the tumor growth. Inflammation caused by dysbiosis could also stimulate carcinogenesis through the selection of the most tumorigenic bacterial strains [67]. DNA damage resulting from the production of pro-oxidative molecules, such as reactive oxygen (ROS) and nitrogen (NOS) species is linked to bacteria as well [68, 69]. Even if relatively young, the field of gut microbiota-CRC interactions is under growing investigation, due to the increasing number of reports evidencing connections with CRC tumorigenesis. Further analyses will add this new perspective to the CRC induction mechanisms.

### CRC therapy

The classical way to treat CRC is surgical resection followed by chemotherapy. It is known that approximately one-fourth of CRC diagnosed patients show synchronous metastasis and almost a half develop metastasis after diagnosis [70]. During last years, drugs able to specifically target dysregulated molecules, with consequent prognosis amelioration, can be used in well-defined cases in association to cytotoxic treatment: this is the case of monoclonal antibodies against VEGF (Bevacizumab) and EGFR (Cetuximab and Panitumumab). No biomarkers are in use for anti-VEGF, whereas predictive biomarkers have been approved and now extensively considered for anti-EGFR therapy in clinical practice [71]. Many studies demonstrated the importance of KRAS and the closely related NRAS genotype in considering the response to anti-EGFR drugs, whose efficacy is mostly observed in KRAS and NRAS wild-type (WT) patients. On the contrary, patients with KRAS or NRAS mutations at the level of exons 2, 3 or 4 do not benefit of anti-EGFR treatment, which can even show detrimental effect when associated to oxaliplatin [72, 73]. However, it is known that a percentage of KRAS or NRAS WT patients is not responsive to EGFR targeted therapy, leaving to hypothesize that additional mediators could be involved in the dysregulation of molecular mechanisms leading to tumor initiation and development. Among them, BRAF and PI3KCA, acting downstream of RAS and involved in the PI3K/Akt/mTOR signaling pathway, have been taken into consideration and are under investigation. With this regard, the 2016 ESMO guidelines recommend BRAF genotyping in KRAS/NRAS WT patients. On the other hand, mutations at the level of PI3KCA seem to predict resistance to anti-EGFR and for this reason the protein is considered as a target for inhibition in clinical trials in progress. Seen in this context, additional potentially actionable genes cannot be excluded. Due to its feature, NGS provides a suitable, fast and cost-effective technology to simultaneously identify multiple genes carrying either described or undescribed mutations which could play an active role in tumor development and, possibly, in driving therapeutic decision making.

## NGS in CRC

### Mutation analysis of actionable genes

Next-generation sequencing provides a fast high-throughput and cost-effective technology with respect to traditional Sanger sequencing to accurately identify mutations in known genes and to provide information of clinical utility [74]. Here we report the results of some of the most recent studies, summarized in Table 2. Peeters et al. [75] investigated the response to panitumumab in patients affected by metastatic CRC (mCRC). Adopting a massive multigene NGS sequencing (Roche GS FLX), the authors analyzed 9 genes in 320 samples and detected mutations in K/NRAS, BRAF, PI3KCA, PTEN, TP53, EGFR, AKT, CTNNB1 genes with frequencies ranging from 60 (TP53) to 1% (AKT). They demonstrated that panitumumab treatment prolonged progression free survival (PFS) in KRAS-WT patients compared to KRAS-mutant patients. Moreover, patients with KRAS- and NRAS/BRAF WT genes showed better response with respect to KRAS-WT and NRAS/BRAF mutant patients. In conclusion, the study provided evidence that NGS can be a suitable method to identify predictive biomarkers. A work by Kothari et al. [76] on 468 CRCs, 77 of them already analyzed by standard test for KRAS in approved laboratories, demonstrated the viability and even the higher diagnostic power of Illumina NGS with respect to standard methods in identifying KRAS mutations, making it possible the detection of lesions with potential clinical impact, not typically evidenced by standard tests. A study by Ciardiello et al. [77] was focused on the analysis of twenty-two cancer-related genes in 182 KRAS exon 2 WT tumor samples from mCRC patients treated with first-line FOLFIRI plus cetuximab. Semiconductor-based NGS revealed one or more (up to 5) gene mutations in 124 out of 182 specimens. KRAS exon 2 mutations were detected in approximately 16% (29/182) of patients, previously classified as wild-type by local laboratory tests, and TP53, KRAS, NRAS, PI3KCA and BRAF were described as more frequently mutated genes as well. Based on NGS gene mutation analysis results, evaluation of ORR and PFS provided information about a group of patients (KRAS and NRAS WT) which could benefit of the FOLFIRI plus cetuximab treatment, confirming the possible NGS use in clinical practice. Bai et al. [78] further highlighted the clinical utility of targeted gene sequencing. They analyzed 91 rectal cancers by Ion Torrent targeted sequencing and found frequent mutations in KRAS, TP53, APC, FBXW7, PI3KCA and, to a lesser extent, in BRAF, CTNNB1, ERBB2 and SMAD4. In addition, they identified associated multiple mutations, mainly involving KRAS and APC or KRAS and TP53. Harlé et al. [79] analyzed NRAS and KRAS in 188 consecutive mCRCs by using GS Junior technology. They identified nine uncommon mutational profiles and showed 4 undescribed nucleotide variants with aminoacid change, focusing the attention on the possibility to detect non-hotspot rare RAS mutations putatively able to impact the response to anti-EGFR. On the other hand, Taieb et al. [80] analyzed by real-time PCR 2559 stage III CRC patients treated with FOLFOX ± cetuximab for hot-spot KRAS and BRAF V600E mutations. Nine-hundred height of them resulted positive. Of the remaining, 1054 were subjected to NGS (Ion Torrent, Ampliseq colon and lung) which allowed uncovering newly diagnosed K/NRAS and BRAF mutations in 227 (21%) and 46 (4.4%) patients, respectively. The authors described neither significant better trend of outcome in BRAF and RAS WT patients treated with FOLFOX plus cetuximab or detrimental effects in those RAS-mutant. However, they identified a clinically relevant 0.76 adjusted hazard ratio value evidenced for disease free survival (DFS) for cetuximab addiction in RAS and BRAF WT patients, suggesting a new randomized trial to test the efficacy of anti-EGFR in this setting and further pointing out the importance NGS analysis. In a very recent targeted exome-sequencing based work on 63 Iranian Shirazi patients, Ashktorab et al. [81] detected and further validated 51 variants in 12 genes by using two NGS platforms (Ion Torrent and Illumina). They showed higher mutation rate of MSH3, MSH6, APC and PI3KCA in Iranian patients, hypothesizing a major role of these genes in CRC and suggesting the adoption of specific informed genetic diagnosis protocol and tailored therapy in this population. Another study [82] confirmed detailed identification of mutations in 138 mCRC, identifying a novel KRAS mutation (KRAS^R68S1^) associated with an aggressive phenotype as well as rare RAS and MET amplification, BRAF and ARAF alterations, PTEN-PI3KCA-AKT pathway mutations associated with poorer prognosis and, possibly, anti-EGFR resistance. The authors described three hypermutated tumors with MSI-H or POLE mutation and ERBB2 amplified tumors (5% of cases) as well, which might benefit of anti-PD-1 or HER2-targeted therapy in absence of RAS/RAF mutations, respectively. Again, a recent study on both cell lines and tumor specimens [83] highlighted the importance of NGS in detecting genes potentially involved in the resistance to anti-EGFR therapy in KRAS wild-type patients. The authors analyzed the response of 7 CRC cell lines to cetuximab as well as primary tumors, liver and lung metastasis from 25 CRC patients treated with cetuximab or panitumumab, by evaluating NGS (Illumina) mutation profiles of 48 cancer-related genes, EGFR and E-cadherin expression. Lack of response to anti-EGFR therapy was associated to ATM mutations and low E-cadherin expression, here described as novel supportive predictive markers. Lee et al. [84] analyzed NTRK1 rearrangements in 74 mCRC and 66 gastric cancer (GC) patients. Two mCRC and one GC TrkA-IHC positive patients were further analyzed by FISH and NGS, which highlighted TPM3-NTRK1 rearrangements. The use of entrectinib, a pan-TRK inhibitor, inhibited cell proliferation of patient-derived tumor cells (PDCs) with rearrangement by TrkA inactivation and downregulation of downstream pathways. In conclusion, this study focuses the attention on novel targeted drugs to be potentially used in presence of specific CRC gene lesions. A very recent work [85] analyzed the intra- and inter-tumor molecular heterogeneity between CRCs and synchronous liver metastasis. The authors studied the genotype of 22 genes (Ion Torrent, Ampliseq colon and lung panel), mainly involved in colorectal tumorigenesis, in primary tumor samples and liver metastasis from 7 KRAS-WT patients, both before and after chemotherapy associated to anti-EGFR. Results showed marked genotype differences by comparing pre- and post-therapy specimens, most probably attributable to tumor cells clones selected by the therapeutic treatment and, at the same time, able to affect the response to therapy. Several studies have further confirmed the NGS improvement in detecting mutation of BRAF, KRAS and EGFR-pathway genes. Fifty-three KRAS exon 2 WT mCRC patients, treated with cetuximab/irinotecan-based chemotherapy, were analyzed by using a panel of 10 genes related to EGFR pathway and NGS semi-conductor technology [86]. This study demonstrated that extensive analysis of EGFR pathway-related genes leads to the identification of variants with predictive value, which could help in individuating non-responders to targeted therapy. In particular, KRAS, NRAS and BRAF mutations are important factors in predicting response to cetuximab in KRAS exon 2 WT patients. Ma et al. [87] analyzed 822 cancers, including CRC, and compared results from Illumina (MiSeq or NextSeq) with those obtained by qRT-PCR based FDA-cleared testing kits, demonstrating that the latter can be now considered not sufficiently accurate. On the contrary, NGS is confirmed a powerful technology, due to the identification of a significant number of KRAS, BRAF, EGFR mutations which are missed by standard tests, but could have clinical relevance.

**Table 2:** NGS to detect lesions in actionable genes

Samples	NGS platform	# of analyzed genes/assay	Mutant genes analyzed	Therapy	Ref.
320 mCRC	454 GS FLX	9 Targeted-seq (KRAS, NRAS, EGFR, BRAF, PTEN, PI3KCA, AKT, TP53, CTNNB1)	K/NRAS, BRAF, PI3KCA, TP53, PTEN, EGFR, AKT, CTNNB1	Panitumumab	Peeters et al. [75]
468 CRC (77 KRAS analyzed)	GAIIx Illumina	1321 Targeted-seq (SureSelect Agilent)	KRAS	Anti-EGFR	Kothari et al. [76]
182 mCRC KRAS ex2 wt	Ion Torrent	22 Targeted-seq (AmpliSeq colon and lung cancer panel)	KRAS (29/182pts) frequently mutant: TP53, RAS, PI3KCA, BRAF	Folfiri/Cetuximab	Ciardiello et al. [77]
91 CRC	Ion Torrent	22	KRAS, TP53, APC, FBXW7, PI3KCA, BRAF, CTNNB1, ERBB2, SMAD4	Anti-EGFR	Bai et al. [78]
188 mCRC	454 GS Junior	2 Targeted-seq (KRAS, NRAS)	KRAS, NRAS	Anti-EGFR	Harlé et al. [79]
1054 stage III CRC	Ion Torrent	22	KRAS, NRAS, BRAF	Folfox/cetuximab	Taieb et al. [80]
63 Iranian CRC	Ion Torrent	15 Targeted-seq (AMER1, APC, ARID1A, BRAF, FBXW7, KRAS, MSH3, MSH6, NRAS, PIK3CA, SMAD4, SOX9, TCF7L2, TGFBR2 and TP53)	MSH3, MSH6, AMER1, APC, BRAF, KRAS, PI3KCA, TGFBR2A, SMAD4, SOX9, TCF7L2, TP53	Suggested informed genetic diagnosis and personalized strategies	Ashktorab et al. [81]
	Illumina	20 Targeted-seq (ACVR2A, AMER1, APC, ARID1A, BRAF, FBXW7, KRAS, MSH2, MSH3, MSH6, NRAS, PIK3CA, POLE, PTEN, SMAD2, SMAD4, SOX9, TCF7L2, TGFBR2 and TP53)			
138 stage IV CRC	FoundationOne method	315 cancer related genes + introns of 28 frequently rearranged genes	RAS, RAF, ERBB2, AKT, PI3KCA, PTEN, MET, POLE	Anti-EGFR, anti-HER2, anti-PD1	Gong et al. [82]
7 CRC cell lines 25 CRC	Illumina MiSeq	48 Targeted-seq (TruSeq Amplicon Cancer)	ATM, E-cadherin	Anti-EGFR	Geiβler et al. [83]
3 mCRC positive for NTRK1 rearrangement	Illumina MiSeq	5 gene rearrangements (NTRK1/2/3, ROS, ALK)	NTRK1	Entrectinib	Lee et al. [84]
54 mCRCs + liver metastasis KRAS ex2 wt	Ion Torrent	22	KRAS, NRAS, TP53, PI3KCA, FBXW7, PTEN	Anti-EGFR	Adua et al. [85]
53 KRAS ex2 WT mCRC	Ion Torrent	20 Targeted-seq (EGFR, KRAS, HRAS, NRAS, BRAF, PIK3CA, AKT1, PTEN, HER2, HER4, TP53)	KRAS, NRAS, BRAF	Anti-EGFR	Hsu et al. [86]

### Novel mutations or less frequently mutant genes

Two studies, based on Sanger sequencing associated to bioinformatics approaches [88, 89], had already highlighted the importance of targeted multi-gene analysis in CRC. They shed light on the tumor heterogeneity and characterized a group of most commonly mutated as well as much larger number of genes less frequently mutated, but involved in several fundamental cell functions, such as transcriptional regulation, adhesion and invasion. As a result, 69 candidate genes with potential oncogenic driver mutations, including both cancer-related and previously uncharacterized genes, were identified, providing interesting insights potentially useful at the clinical level. Later, NGS-based studies provided interesting data (Table 3). Han et al. [90] analyzed 60 normal/tumor tissue pairs from colorectal adenocarcinoma patients by NGS (GAIIx Illumina) and considered 183 cancer-related genes, known to predict response, therapeutically targetable, involved in major signaling pathways. The presence of 232 different somatic point mutations, 166 novels and 66 known, as well as copy number variations was highlighted. APC, TP53, KRAS were the most mutated genes and the ErbB pathway was described as the most affected, providing data about the usefulness of this method for clinical application. By using HiSeq whole-genome sequencing approach, Shanmugam et al. [91] analyzed a small number of refractory metastatic CRCs to possibly identify new therapies, and described mutations of several interesting genes. Beyond KRAS, APC, PI3KCA and TCF7L2 mutations, they discussed the significance of a lesion of INPPL1, a gene involved in PI3K/AKT signaling pathway. They identified the E567G aminoacid substitution in its SHIP2 protein product, a phosphatase which converts PIP3 to PIP2, negatively regulating PI3K/AKT signaling. The authors demonstrated that INPLL1 in vitro knock-down abolished cell growth, leaving to hypothesize that gene mutations might induce gain of function leading to cancer promotion and providing insights about its possible actionable role. In a work on 653 routine CRC, Malapelle et al. [92] analyzed a panel of 22 significant genes by using semiconductor-based technology. Besides the most frequently mutant genes, they described 12 additional genes carrying at least one mutations, highlighting potential actionable molecules in CRC. Interestingly, they identified for the first time in CRC the p.L1196M mutation on ALK, whose protein product induces high resistance to the RTK inhibitor Crizotinib in lung cancer. In addition, they described AKT1, STK11, ERBB2, ERBB4, MAP2K1, NOTCH1 infrequent mutations (0.9–0.2%) as well. Talseth-Palmer et al. [93] analyzed by HiSeq a panel of 22 genes involved in MMR pathway in HNPCC and EC (endometrial cancer) patients and identified five exonic indels, 42 non-synonymous nucleotide substitutions and one intronic mutation. In CRC patients, one variant was classified likely pathogenic (MSH2, c.186_187 dup), two with uncertain relevance (EXO1, c.2212-1G > C; POLD2, c.203G > T) and 36 with unknown significance (in EXO1, LIG1, MLH1, MLH3, MSH3, MSH6, PMS1, PMS2, POLD1, RPA1 genes). The novel POLD2 c-203G > T variant was associated to the rare EXO1 c.2212-1G > C in a patient. Stadler et al. [94] analyzed (HiSeq) panels of 341 or, updated, 410 cancer associated genes in 224 CRCs with available IHC staining for MMR. Among them, 193 specimens with less than 20 mutations were MMR-proficient, whereas 28 out of 31 showing more than 20 mutations were MMR-deficient (MMR-D). The remaining three samples evidenced more than 150 mutations and an ultramutator phenotype with somatic alterations at the level of POLE exonuclease domain (P286R) as well as additional mutations in more frequently mutant genes (KRAS, TP53, PI3KCA, KIT). The NGS analysis was cost effective, able to characterize both MMR and RAS/BRAF mutations and to provide a cutoff value for a “mutational load” which could be considered a very sensitive method to screen MMR-D cases. Finally, NGS data collected from more than 9600 mCRC patients were analyzed to assess the presence and role of non-V600 BRAF mutations [95]. Non-V600 mutations were found in 2.2% (208) of all patients tested and accounted for 22% of all detected BRAF mutations. They seem to be correlated to a clinically distinct CRC subtype with better prognosis.

**Table 3:** NGS to detect novel mutations or less frequently mutant genes

Samples	NGS	# analyzed genes	Novel mutations/less frequently mutant genes	Ref.
60 pairs normal/CRC	GAIIx Illumina	183 Targeted-seq (custom panel)	166-point mutations 25 indels	Han et al. [90]
4 mCRC	Illumina HiSeq	Whole genome (NEBNext DNA New England)	INPPL1 p.E567G	Shanmugam et al. [91]
77 CRC	Ion Torrent	22 Targeted-seq (ampliseq colon and lung)	ALK p.L1196M/AKT1, STK11, ERBB2, ERBB4, MAP2K1, NOTCH1	Malapelle et al. [92]
14 HNPCC, 12 EC, 2 LS	Illumina HiSeq	22 Targeted-seq (MMR custom panel)	POLD2, EXO1	Talseth-Palmer et al. [93]
224 CRC	Illumina HiSeq	341/410 cancer associated genes (Msk-impact)	POLE p.P286R	Stadler et al. [94]
9643 mCRC	Ion Torrent	50 Targeted-seq (Ampliseq cancer hotspot)	Non-V600 BRAF	Jones et al. [95]

### NGS transcriptomics analysis

Post-transcriptional events in CRC gained attention and interesting results have been collected by NGS (Table 4). Some scientists took advantage of NGS technology to sequence human CRC mRNAs to verify RNA changes, such as alternative cleavage and polyadenylation (APA), during CRC progression [96]. They analyzed APA in 15 CRC patients by Illumina platform and found many genes with progressive APA changes. Results were further validated in 50 patients, and five normal/tumor tissue pairs. Three genes (PPIE, DMKN, PDXK), with significant modifications by comparing normal mucosa/adenoma/carcinoma, were proposed as potential biomarkers. In another study, Banky et al. [97] analyzed by GS Junior the alternative splicing pattern (ASP) of CD44, a gene associated with cancer and metastasis, in different human CRC cell lines. They provided a list of CD44 isoforms expressed by CRC, but absent in normal tissue. Furthermore, the CD44 isoforms’ expression pattern remained constant both in CRC cells and primary and metastatic cancer xenografts. Moreover, they found high levels of CD44 v3 and v6 variants co-expression in tumor cells more prone to give raise to metastasis, suggesting a specific role of these splicing variants in CRC development and progression. Wu et al. [98] performed high throughput RNA-seq (Illumina) to compare CRC, adjacent non-tumor and distant normal tissues obtained from the same patient. The study revealed differentially expressed genes as well as alternative splicing, novel and fusion transcripts. Among the latter, the authors validated the tumor-restricted PTGFRN-NOTCH2. Due to the role of NOTHC2, considered a prognostic predictor linked to the “tumor differentiation status” in CRC, the authors hypothesized that this fusion transcript could have a dominant negative effect on normal cell development.

**Table 4:** NGS in transcriptomics analysis

Samples	NGS	Application	Interesting genes	Ref.
15 CRCs	Illumina HiSeq (3′-seq library)	APA changes	PPIE, DMKN, PDXK	Morris et al. [96]
3 CRC cell lines	Roche GS Junior (primers framing CD44 variable region)	CD44 ASP	CD44 CRC specific isoforms	Bànky et al. [97]
1 stage III CRC patient (distant, adjacent mucosa and cancer)	Illumina GAIIx (TruSeq RNA)	RNA-seq (novel and fusion transcripts, alternative splicings)	List of 14 genes with cancer-associated differential-splicing, PTGFRN-NOTCH2 fusion product	Wu et al. [98]
70 CRCs/normal pairs	Illumina HiSeq (TruSeq RNA)	RNA-seq	RSPO2/3 fusion transcript	Seshagiri et al. [99]
20 CRC cell lines	Illumina (ChIP-seq)	RNA-seq (genes involved in irinotecan resistance)	20 top-genes CDC20, CTNNAL1, FZD7, CITED2, ABR, ARHGEF7 and RNMT validated by qPCR	Li et al. [100]
21 CRC cell lines	Illumina (ChIP-seq)	RNA-seq (genes involved in oxaliplatin resistance)	58 top-genes 15 further validated by qPCR (e.g. HNF1A, NOTCH1, FZD5, KCND1, FDZ2)	Li et al. [101]
175 CRCs/normal tissue pairs	Illumina HiSeq (TruSeq)	RNA_seq (CRC vs. normal differentially expressed genes)	1138 up-regulated 695 down-regulated	Slattery et al. [102]
HCT116 CRC cell line	Illumina HiSeq	RNA-seq (SNP profiles from single cell and bulk CRC)	Genes in WNT, PI3KCA, p53, TGF-β, MMR pathways, fusion transcripts	Chen et al. [103]
5 paired primary, mCRC, normal colon and liver tissues	Illumina HiSeq	RNA-seq (comparative transcriptome analysis)	Similar expression profiling in CRC and metastasis, RNF43-SUPT4H1 fusion transcript frequent in primary CRCs	Lee et al. [104]
217 CRCs/normal mucosa pairs	Illumina HiSeq (TruSeq)	RNA-seq (oncogenes, tumor suppressors’ differential expression)	22 tumor suppressor genes and 27 oncogenes significantly differentially expressed in CRC vs. normal mucosa	Slattery et al. [105]

Wnt/APC pathway is altered in the most of CRC genomes, clearly demonstrating how this signaling is central in colorectal carcinogenesis. Its importance is further highlighted by the work of Seshagiri et al. [99] who analyzed more than 70 primary tumor pairs to characterize exome, transcriptome and copy number variation. They detected high numbers of mutations in many genes and identified 23 significantly mutated genes. By RNA-seq approach (Illumina), they discovered R-spondins (RSPO-2 and -3) fusion transcripts in 10% of primary CRCs. The R-spondins are secreted proteins able to potentiate canonical Wnt signaling. The presence of these aberrant transcripts seemed to mutually exclude APC and CTNNB1 mutations and, at the same way, to enhance WNT signaling, thus suggesting an alternative mechanism for WNT pathway aberrant activation. Li et al. [100] used 20 CRC cell lines to identify 20 top genes responsible for resistance/sensitivity to irinotecan and further validated 7 of them (CDC20, CTNNAL1, FZD7, CITED2, ABR, ARHGEF7 and RNMT) by qPCR in two resistant and sensitive CRC cell lines. Major differences, most probably indicating the involvement of them in the response to irinotecan, were detected in CTNNAL1, FZD7, CITED2 genes, overexpressed in the resistant cell line, and in ARHGEF7, overexpressed in the sensitive. A similar work [101], aimed to test the sensitivity to oxaliplatin, highlighted more than 50 top genes whose 15 were further validated: among them HNF1A, NOTCH1, FZD5 were negatively correlated to oxaliplatin resistance, and KCND1, FDZ2, positively correlated. The above-mentioned works opened to novel biomarkers predicting the response to two of the most used chemotherapeutic drugs for CRC. Slattery et al. [102] analyzed 175 tumor/normal tissue pairs from CRC patients and further processed almost 2000 differentially expressed genes to identify key pathways (cell signaling and growth). They concluded that having more dysregulated pathways is associated with a better prognosis, supporting this evidence with the observation that stage 1 patients have more dysregulated genes than those stage 4, probably due to the activation of more key events to arrest tumor progression. Chen et al. [103] performed SNP analysis by using RNA-seq data obtained by single-cell and bulk colorectal cancer cells. They assessed that single-cell RNA-seq is a powerful method to replicate the results of bulk analysis and, in addition, can reveal individual cell features not detectable in bulk-sample SNP analysis. After SNPs identification, GO (Gene Ontology) elaboration was performed and a list of cancer-related genes as well as pathway enrichment and fusion gene analysis were obtained. Mutations and fusion transcripts were identified at the level of genes involved in several key signaling pathways, including TGF-β, p53, PI3KCA, WNT and MMR. Lee et al. [104] showed by Illumina RNA-seq that gene expression patterns were highly similar in paired primary CRCs and liver metastasis, but, at the same time, identified fusion transcripts which were differentially expressed and could help to distinguish between primary tumor and metastasis. Among them, they described RNF43-SUPT4H1 in primary CRC, whose knock-down showed growth inhibitory effect. Slattery et al. [105] demonstrated the existence of co-regulatory networks involving tumor suppressor genes, oncogenes and miRNAs which might interact and play a role in regulating mechanisms of oncogenesis. To this aim, they analyzed 217 CRCs paired to normal mucosa and detected 22 differentially expressed tumor suppressor genes: 10 were up-regulated (FAM123B, RB1, TP53, RUNX1, MSH2, BRCA1, BRCA2, SOX9, NPM1, and RNF43), six downregulated (PAX5, IZKF1, GATA3, PRDM1, TET2, and CYLD), four were associated with MSI cancers (MLH1, PTCH1, and CEBPA down-regulated and MSH6 up-regulated) and two linked to MSS tumors (PHF6 and ASXL1 up-regulated). In addition, thirteen of those tumor suppressor genes were associated with 44 miRNAs. Among the oncogenes, 27 were dysregulated: 14 downregulated (KLF4, BCL2, SSETBP1, FGFR2, TSHR, MPL, KIT, PDGFRA, GNA11, GATA2, FGFR3, AR, CSF1R, and JAK3), 7 up-regulated (DNMT1, EZH2, PTPN11, SKP2, CCND1, MET, and MYC), 5 dysregulated in MSI (FLT3, CARD11, and ALK hypo-expressed, IDH2 and HRAS hyper-expressed), 1 up-regulated in MSS (CTNNB1). RNA-seq datasets available on the internet have been also used for in silico studies. Snezhkina et al. [106] analyzed Cancer Genome Atlas RNA-seq datasets obtained from CRC and normal tissue pairs. They found more than one thousand alternative mRNA isoforms involved in cell metabolism, identifying 7 genes whose alternative transcripts were differentially expressed in CRCs, even though their overall expression was not different. Eight differentially expressed isoforms encoded by OGDH, COL6A3, ICAM1, PHPT1, PPP2R5D, SLC29A1, and TRIB3, further validated by qRT-PCR, resulted up-regulated in CRC, providing evidence about tumor specific alternative transcripts of genes involved in metabolism, which putatively play a role in CRC.

### Epigenetics analysis

Next-gen technology has been also applied to epigenetics studies, principally focused on evaluating the methylation level or microRNAs expression in CRC. Some valuable profiling researches were conducted to sequence the methylome of CRC samples, being DNA methylation of several genes reported in CRC [107]. Hansen et al. performed a study by using Illumina platform (whole-genome bisulfite seq) proving the existence of blocks of hypomethylated regions encompassing a half of the genome [108]. They also reported the presence of certain cancer-specific differentially methylated regions characterized by high level of gene expression variability and demonstrated different degrees of methylation by comparing colon normal tissues, adenomas and carcinomas. Another study with the same platform confirmed these findings documenting that, in many CRC, genome regions of focal hypermethylation are concentrated into CpG islands and reside inside large hypomethylated blocks [109]. These sequences correspond to late replication and attachment to the nuclear lamina regions in human cell lines. Exciting possibilities offered by next-generation technology are clear also in the context of microRNA (miRNA) research, small non-coding RNA molecules able to post-transcriptionally regulate gene expression, involved in the control of many fundamental cell functions and considered as promising therapeutic targets and/or biomarkers [110, 111]. To this aim, a study focused on the differential expression of miRNAs between paired normal and tumor colon samples is explanatory. By using the Illumina GAIIx system, the authors described the discovery of 16 dysregulated miRNAs, previously undescribed in CRC. Among the most interesting hyper-expressed, they showed miR-549, located within the locus of KIAA1199 gene, already reported as strongly up-regulated in many colon carcinomas [112]. They suggested the possibility of using miR-549 as early CRC surrogate biomarker, since it could be co-transcribed with KIAA gene. Later, Rohr et al. [113] analyzed, with Illumina GAII, miRNA and mRNA profiles from normal, primary tumor and metastasis tissues and identified 4 miRNAs (MiR-1, -129, -497, -215) as largely dysregulated in cancer. The authors performed a system-biology simulation aimed to analyze the effects of miRNA-1 as a putative therapeutic option, providing an in silico model for personalized cancer treatment. Recently, Koduru et al. [114] further confirmed the results of Rohr et al. by performing bioinformatics analysis on the same data. They identified 13 aberrantly expressed miRs in cancer and metastasis throughout the progression of the disease and evidenced very similar miRs’ levels between primary tumors and metastasis. Neerincx et al. [115] analyzed miRNA expression profiling (HiSeq) in 220 fresh-frozen samples from paired primary colorectal cancers, metastases and non-tumor tissues. The study revealed approximately 222 miRs able to differentiate primary tumors and metastasis from non-tumor tissues. Among them, the most specific appeared to be miR-21 and miR-92a, already described as putative CRC early diagnosis circulating biomarkers. On the contrary, only eight miRNAs, either already known or novel candidates (miR-320b, miR-320d, miR-3117, miR-1246, miR-663b, chr 1-2552-5p, chr 8-20656-5p and chr 10-25333-3p), were differentially expressed in primary tumors compared to metastasis, indicating a comparable profiling which could be, however, of clinical utility to predict prognosis or response to therapies.

### Other NGS directions in CRC

Recently, scientific literature was enriched with papers demonstrating that NGS can be suitable for the analysis of circulating tumor cells or cell-free DNA. This is a very interesting field with enormous potential to better understand the biological mechanisms at the base of cancer and to identify new diagnostic, prognostic or predictive biomarkers. With this regard, Heitzer et al. [116] demonstrated the possibility to use NGS technology (Illumina MiSeq) to identify mutations in single circulating tumor cells (CTCs) extracted from mCRC patients. A panel of 68 colorectal cancer-associated genes was used. Specifically, the mutational spectrum was compared in primary tumor, metastases and CTCs. The paper showed that some mutations in relevant genes (APC, KRAS, PI3KCA), previously found only in CTCs, could be uncovered at subclonal level also in the main tumor and in metastases of the same patient, thanks to the use of specific algorithms. NGS feasibility in this context is also confirmed by another recent work [117], where the authors show the possibility to detect with high sensitivity and specificity 568 mutations within six genes (EGFR, K/NRAS, BRAF, cKIT, PDGFRa) (SiRe panel) in cell-free DNA obtained from serum and blood samples from patients affected by mCRC, non-small cell lung cancer (NSCLC) and melanoma. The study was performed by semiconductor- based PGM. If confirmed, these evidences would allow to move towards less invasive tests to follow the progression of the disease and solve some practical issues, like tumor tissue unavailability. Although reiterating NGS feasibility, other studies pose some limits, particularly with respect to the overall clinical sensitivity [118]. Some of them suggest implementing NGS with mutant allele enrichment or use digital PCR to enhance reliability [119].

As above mentioned, the role of microbiome in CRC is a field of increasing interest. Encouraging data came from studies aiming to connect an altered gut microbiota with enhanced risk of CRC development. A research on fecal bacterial DNA in 94 healthy subjects and 47 CRC patients established that patients harboring tumors were characterized by reduced microbial community diversity [120]. In this case, CRC subjects seemed to have lower abundance of *Clostridia* and higher expression of pro-inflammatory genus such as *Fusobacterium* and *Porphyromonas*. Later, the same authors further demonstrated some inter-relations between fecal microbiota and metabolome, confirming the possible involvement of the latter genus in CRC [121]. NGS application resulted to be crucial for genetic sequencing of bacterial genomes in this context.

## Closing remarks: NGS pros and cons

Next-generation sequencing technology can be considered as the future of high-throughput data analysis and genomic sequencing, providing a method to obtain high-throughput data with sensitivity and specificity. It offers the possibility to have massive parallel multigene sequencing in few hours, with significant time and cost reduction, by using very low amount of nucleic acids [122]. This is of great advantage for CRC and, in general, solid tumors clinical application, since the only available material is often derived from biopsied specimens. Of note, a work of Goswami et al. [123] described a list of pre-analytical factors to be considered to increase NGS success rate in this context, such as the quantity of DNA (it should be more than 10 ng), tumor cellularity, resection procedures and biopsied tumor dimensions. Concerning the cost, data from the NHGRI-funded genome sequencing groups indicate that a whole-exome sequence can be produced with approximately 1000 dollars [122]. The clear potential of these technologies is to enhance sequencing power, leading to more complete definition of the genomic landscape. This is important especially for the study of complicated diseases, such as cancer, because it permits to obtain a wider view of the genotype. Next-gen techniques can provide valuable data about mutational status, copy number variations, transcriptomics and epigenetics with the opportunity to combine current available single genetic tests into a unique test able to detect multiple variants. Great attention should be focused on the possibility to generate databases where the sequencing information of single patients can be stored, resulting accessible for future use by clinicians in terms of retrospective analysis and, possibly, therapeutic decisions. This is a fascinating scenario proposed in a review by Kamalakaran et al. [124]. But NGS technologies show also some challenging issues related to technical, clinical and regulatory/legal fields. Many efforts are currently in progress to adopt standardized procedures and initiatives for quality management. Standard procedures for sequencing workflows, at the pre-analytical, analytical and post-analytical level, standard procedures for sequence data handling, processing and storage have been proposed by companies, organizations and societies [125, 126]. Recent published data by Ashktorab et al. [127] evidenced significant variability between two among the most common platforms, Illumina and Ion Torrent, in their calling for nucleotide variants in CRC. Therefore, validation of NGS data by using additional sequencing methods, such as a second NGS platform or Sanger sequencing, is strongly suggested and can greatly improve the accuracy of the results obtained. Different NGS approaches have been conceived to shed light on colorectal carcinogenesis and to possibly isolate novel variants of clinical interest. For this purpose, the available tests progressed from hotspot in actionable gene, cancer-related and even more exhaustive gene panels. Free resources on the internet, such as the Genetic Testing Registry (GTR), are available to provide a central location for voluntary submission of genetic test information by providers (www.ncbi.nlm.nih.gov/gtr). With this regard, an important issue is about consensus panels to use for CRC NGS diagnostics. ESMO guidelines recommend testing exons 2, 3, 4 of KRAS and NRAS, essential for driving therapeutic decisions, and BRAF exon 15. In this review, we reported CRC NGS analyses directed to a variable number of cancer-related genes, ranging from more than 1300 to few genes, with common use of commercially available panels (Ampliseq, TruSeq). Among the genes included in the different panels used, KRAS, NRAS, BRAF, PI3KCA, EGFR are the most shared, due to their relevant role in CRC pathogenesis and treatment. Larger panels require longer time for procedure execution and higher cost, but, on the other hand, provide more extensive knowledge about mutational status, with the possibility to identify new genes of clinical utility for CRC management. In our opinion, KRAS, NRAS, BRAF and PI3KCA gene panel should at least be used. Also, issues related to the amount of NGS data should be addressed. These systems generate many Gb of data to analyze. It is challenging for bioinformaticians to organize and interpret these immense quantities of data. They need to use more powerful computers with advanced algorithms to perform analyses, with consequent problems in terms of both economic and human resources for small medical units or laboratories. Different bioinformatics tools are supplied with the NGS platforms, but additional measures are necessary to ameliorate good data generation and interpretation. Many physicians and researchers are actively promoting courses focused on updating the knowledge in the field. More genotypic and phenotypic data will be also needed to assess the impact of genomic variants in healthy and ill patients. Dong et al. [128] suggested a solution for these issues consisting in networking and partnership to have a solid panel of evidence-based results towards an improved understanding of the data. Furthermore, genetic sequencing raised regulatory concerns about its use in health structures. Regulation and approval of the procedure is essential as well as uniformity about methods and standards definition. Data storage and privacy should be standardized to avoid possible discriminations in the healthcare context: for example, in the field of health insurance. United States approved in 2008 the Genetic Information Nondiscrimination Act (GINA) to prevent these problems and legally regulate genetic testing data for public use.

## Conclusions

Advances in technology made it possible to improve technical skills in nucleic acids sequencing. From the initial results of Sanger technique to the actual next-generation sequencing, a lot of work has been done trying to consider the “individual variability” to move to the “personalized medicine”. Currently, NGS technology stands out as one of the most powerful and effective approach for fast DNA/RNA sequencing. In cancer research, many scientists are striving to exploit this technology at its best and some laboratories are starting to show exciting data, especially in the case of CRC. However, it should be noted that the amount of data in the field is still limited. Additional studies are required to obtain more significant reliability of this technology for clinical application. This means that, maybe, a proper optimization to discover the whole potential of these platforms could be achieved in some years from now. The concept of NGS use in clinical routine is challenging, since these tools produce good results in terms of detecting clinically relevant mutations, but often are not able to repeat these successful performances when wider regions of the genome are subjected to analysis. Specific improvements in quality control methods (i.e. the identification of correct quality parameters) could greatly help to overcome these problems. Additionally, the introduction of NGS technology as clinical tool will require for sure measures for process standardization, data handling and interpretation. Greater attention should be paid to the work of bioinformaticians and biostatisticians for the analyses of the massive quantity of data these systems will generate. Clinical challenges are principally based on obtaining accurate data which can be also easy to interpret, by taking into consideration critical issues related to somatic mutation detection in CRC and solid tumors, foremost the accuracy in identifying lesions with very low allelic frequencies. With this regard, innovative approaches for alignment, assembler and variant calling should be devised to augment the accuracy of the entire NGS workflow. Still today, bioinformatics approaches are agnostic about the disease under study and do not embed in their computation the knowledge specific to the disease or gene under analysis, as instead do the scientists in their evaluations. In this direction, a disruptive approach would be to devise new bioinformatics methods that are aware of the pathology and disease the scientists are looking for and add this knowledge while executing their analysis. In our opinion, this would considerably increase the accuracy of NGS results. At the same level, investments should be made for appropriate education and formation of clinicians about the interpretation of the clinical significance of the data obtained.

In conclusion, NGS technology surely represents a giant step forward in the direction toward personalized medicine against CRC, but further analyses are necessary to reach more complete results and higher level in our view of the big picture.

## Abbreviations

NGS: next generation sequencing
CRC: colorectal cancer
mCRC: metastatic colorectal cancer
FAP: familial adenomatous polyposis
MAP: MUTYH-associated polyposis
HNPCC: hereditary nonpolyposis colorectal cancer
MMR: mismatch repair
CIN: chromosomal instability
MSI: microsatellite instability
CIMP: CpG islands methylator phenotype
ROS: reactive oxygen species
NOS: nitrative oxygen species
VEGF: vascular endothelial growth factor
EGFR: epidermal growth factor receptor
FOLFOX: folinic acid, fluorouracil, oxaliplatin
FOLFIRI: folinic acid, fluorouracil, irinotecan
ZMW: zero mode-waveguide
EMT: epithelial-to-mesenchymal transition
3′UTR: 3′untranslated region
CTC: circulating tumor cells
